# Estimating the effect of active detection and isolation on *Clostridioides difficile* infections in a bone marrow transplant unit

**DOI:** 10.1017/ice.2023.37

**Published:** 2023-10

**Authors:** Kelly A. Reagan, David M. Chan, Ginger Vanhoozer, Gonzalo Bearman

**Affiliations:** 1 Department of Mathematics and Applied Mathematics, Virginia Commonwealth University, Richmond, Virginia; 2 Division of Infectious Diseases, Virginia Commonwealth University, Richmond, Virginia

## Abstract

**Objective:**

To model the effects of active detection and isolation (ADI) regarding *Clostridioides difficile* infection (CDI) in the bone marrow transplant (BMT) unit of our hospital.

**Setting:**

ADI was implemented in a 21-patient bone marrow unit.

**Patients:**

Patients were bone marrow recipients on this unit.

**Interventions:**

We compared active ADI, in which patients who tested positive for colonization of *C. difficile* before their hospital stay were placed under extra contact precautions, with cases not under ADI.

**Results:**

Within the BMT unit, ADI reduced total cases of CDI by 24.5% per year and reduced hospital-acquired cases by ∼84%. The results from our simulations also suggest that ADI can save ∼$67,600 per year in healthcare costs.

**Conclusions:**

Institutions with active BMT units should consider implementing ADI.

*Clostridioides difficile* infections (CDIs) are one of the most common healthcare-associated infections in the United States.^1^ The Centers for Disease Control and Prevention (CDC) reports that nearly half a million CDIs occur in the United States each year.^2^ In a study from 2021, estimates indicated that CDIs nearly quadruple hospitalization costs.^3^ The cost of treating CDIs has been estimated at $1.5 billion annually in the United States.^4^ *C. difficile* is an anaerobic bacterium that produces spores and toxins that lead to diarrhea and colitis. Many people live with *C. difficile* bacteria in their gut as a part of their natural microbiome. However, when the gut is disturbed, *C. difficile* bacteria can produce harmful toxins and cause an infectious syndrome. Symptoms of CDI include severe watery diarrhea, fever, stomach tenderness, loss of appetite, and nausea.^2^

Cases of CDI can be classified upon their origin: community-acquired or hospital-acquired (hospital-onset). If a patient develops symptoms of CDI within 48 hours of admission and their last hospital discharge was at least 12 weeks prior, then their case is classified as community-acquired CDI. However, if a patient has been in the hospital for >48 hours, then the CDI case is considered to have been hospital acquired.^5^ A patient may have acquired CDI from the community through outpatient healthcare institutions, receiving antibiotics through the outpatient healthcare institution, or ingesting contaminated food or water.^5^

Immune-compromised individuals, elderly people, and patients prescribed antibiotics are more susceptible to getting CDI than the general population.^1^ Immune-compromised individuals lack the ability to fight off harmful bacteria, such as toxic *C. difficile* spores. Elderly people are more susceptible to CDI due to frequent healthcare visits and physiological changes to their gut.^6^ Antibiotics alter the patient’s microbiome, which can trigger otherwise unproblematic *C. difficile* to produce toxins. Patients in the bone marrow transplant (BMT) unit are prone to CDI because they are immune compromised and are prescribed antibiotics during their treatment.^7^

*Clostridioides difficile* is transmitted when infectious and asymptomatically colonized (or newly colonized) patients shed *C. difficile* spores into the environment that can then enter a susceptible patient’s body through the mouth.^8,9^ A patient who has ≥3 loose stools within 24 hours is tested for toxigenic *C. difficile* to determine whether the patient has CDI and not another diarrhea-causing condition.^1^ When the patient is symptomatic, they are placed under contact precautions. Contact precautions include hand washing, wearing gloves and gowns, requiring patients to stay in an isolated room, and disinfecting the room and equipment with sporicidal disinfectants.^10,11^

Instead of only testing patients with symptoms, active detection and isolation (ADI) can be implemented in which patients are tested before entering the hospital to determine whether they are colonized by *C. difficile*.^12^ When a patient tests positive by polymerase chain reaction (PCR) testing upon admission, that patient is isolated and placed under contact precautions for the remainder of their hospital stay.^13^

ADI is not always implemented due to costs and being more resource intensive than testing a patient only when they are symptomatic.^14,15^ Additional resources needed for ADI include performing additional tests, rooms for isolating patients, healthcare workers to administer the test, and protective equipment for healthcare workers. Resistance to implementing ADI includes feedback from patients about increased isolation, depression, and/or anxiety; prolonging the patient’s stay; and increased wait time in emergency departments.^15^

Overall, ADI has been shown to reduce the incidence of CDI.^7,13,16,17^ Particularly vulnerable wards in the hospital, such as the BMT unit, can benefit from ADI.^7^ In this study, we used mathematical modeling to describe how ADI decreases CDIs and to quantify the costs associated with implementing ADI within the BMT unit.

## Methods

### Mathematical model

We focused our model on the BMT unit at Virginia Commonwealth University Medical Center. This BMT unit has a capacity for 21 patients in individual rooms. Because patients acquire *C. difficile* indirectly by ingesting *C. difficile* spores from the environment, our model incorporates patient interactions with a contaminated environment. Due to the small number of patients in the BMT unit, we utilized an agent-based model (ABM) to simulate the interactions between the patients and the environment. The environment encompassed the room, healthcare workers, and shared equipment, among other things.

In the model, agents were patients, and the environment comprised the BMT unit and healthcare workers. The level of contamination in the environment was determined by estimating the level of spores shed by colonized and infectious patients. When a patient was discharged, we assumed that the patient’s room was disinfected and that another patient was admitted into the room. Thus, the level of *C. difficile* contamination within the environment decreased when a patient was discharged.

**Fig. 1.**
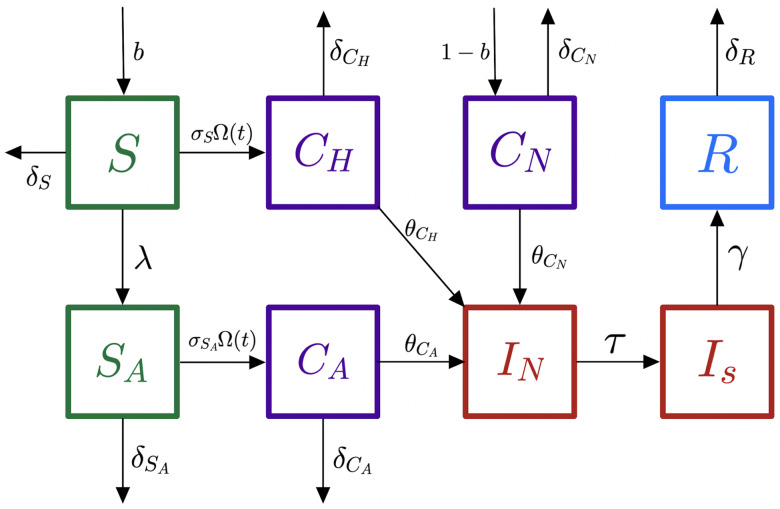
Model diagram for non-ADI model with patient states. Note. susceptible, *S*, susceptible on antibiotics, *S*_*A*_, asymptomatic colonization by environment, *C*_*H*_, asymptomatic colonization by antibiotics, *C*_*A*_, admitted with asymptomatic colonization, *C*_*N*_, infectious, not screened yet, *I*_*N*_, infectious, screened, *I*_*S*_, and recovered, *R*. The arrows indicate a probability of transitioning to the next class.

**Fig. 2.**
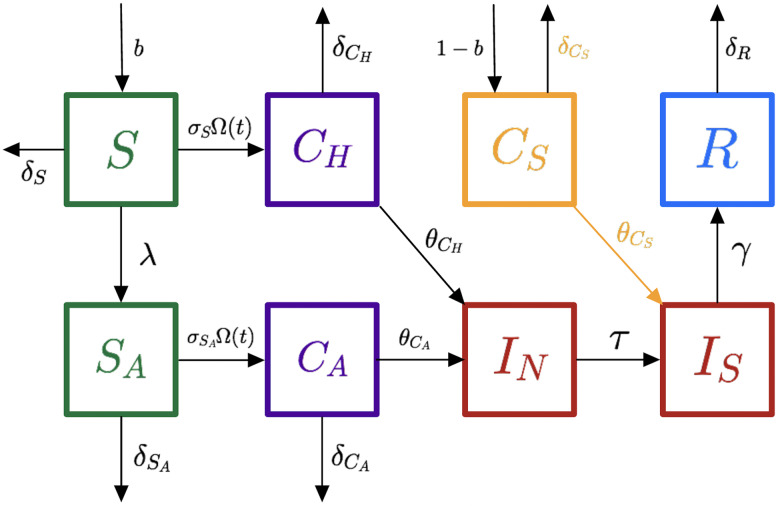
Model diagram for ADI-model when ADI is implemented. The differences between the non-ADI model and the ADI model are highlighted in gold.

Although patients do not directly interact with one another, the patients’ contributions to the contamination of the environment causes *C. difficile* to spread. Patients in the BMT unit are at high risk for CDI due to long hospitalizations and high antibiotic use and because chemotherapy negatively impacts a patient’s intestinal health.^7^ Two agent-based models, an ADI model and a non-ADI model, were constructed to measure the outcomes of implementing ADI on the transmission of *C. difficile*. The non-ADI model considered the practice of testing only symptomatic patients (Fig. 1), and the ADI model considered the process of ADI (Fig 2).

For the environment, *P*(*t*) is an estimate of the amount of contamination in the environment, and Ω(*t*) is the proportion of environment that is contaminated with *C. difficile* spores. *P*(*t*) is defined as follows:

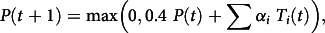

where *i* ∈ {*C*_*A*_, *C*_*H*_, *C*_*N*_, *C*_*S*_, *I*_*N*_, *I*_*S*_, *R*, *D*}, *α*_*i*_ ∈{−1,0,1,2}, and *T*_*i*_ is the number of spores shed by class *i*. The contribution of patients shedding spores is quantified by α_*_, where * denotes the state of the patient. Taking the maximum of zero and the summation ensures that *P*(*t*) is nonnegative. A positive α_*_ value indicates that the class added spores to the environment and a negative value removed spores from the environment. Spores are eliminated when a patient is discharged (eg, *α*_*R*_ = −1).

Also, Ω(*t*) utilizes the total contribution of infectious spores by colonized and infected patients and is given by the following formula:

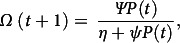

where *η* approximates the threshold point where the environment becomes more toxic, and *ψ* determines how quickly the environment transitions to being more toxic.

The agents consist of the set of patients in the BMT Unit. Patients can be in 1 of 8 different compartments on any given day. For the non-ADI model, patients can either be admitted into *S*, if they are not colonized, or to *C*_*N*_, if they are colonized (Fig. 1). We assume that the general population is colonized at a rate of (1 – *b*).

From *S*, a patient can be prescribed antibiotics and move into the *S*_*A*_ class, or they can become colonized from exposure with a contaminated environment and transition into the *C*_*H*_ class. Every patient can be discharged from class *κ*, *κ* ∈ {*S*, *S*_*A*_, *C*_*H*_, *C*_*A*_, *C*_*N*_, *R*}, at a rate of *δ*_*κ*_, unless they are infectious. Patients are transitioned from susceptible to colonized at the rate *σ*_*z*_Ω(*t*), *z* ∈{*S*, *S*_*A*_}.

From *S*_*A*_, if a patient becomes colonized, the patient moves into *C*_*A*_. Any colonized patient, *C*_*A*_ *, C*_*H*_, or *C*_*N*_, can develop CDI and transition into *I*_*N*_. Patients in *I*_*N*_ have not yet been placed under contact precautions. Once a patient is in *I*_*N*_, they can only transition into the *I*_*S*_ class. While a patient is being treated for CDI in *I*_*S*_, they stay in *I*_*S*_ with additional contact precautions. When they recover, they move to *R*. Once a patient is in *R*, they remain there until they are discharged.

Incorporating ADI alters 1 patient compartment and 1 transition between compartments in the model. With ADI implemented, before patients are admitted, they are tested for colonization of *C. difficile* bacteria and are immediately placed under contact precautions in *C*_*S*_ if they test positive. Otherwise, if they are not colonized, they are placed in *S* (Fig. 2).

The goal of the research is to determine how ADI reduces cases of CDI, and to track hospital-acquired and community-acquired infections. Patients that transition from either *C*_*H*_ to *I*_*N*_ or *C*_*A*_ to *I*_*N*_ count as hospital-acquired CDIs. The number of community-acquired cases of CDI is calculated by counting the number of new patients entering *I*_*N*_ from *C*_*N*_ in non-ADI model. In the ADI-model, the number of community-acquired cases was calculated by adding all of the patients who transition from *C*_*S*_ to *I*_*S*_.

Both models are used to quantify the cost of implementing ADI to compare the cost of testing patients only when they are symptomatic. In the non-ADI model, any time a patient entered *I*_*N*_, a test was used. In the ADI model, all admitted patients were tested in addition to any patient who entered *I*_*N*_ from {*C*_*H*_ *,C*_*A*_} or *I*_*S*_ from *C*_*S*_. Other costs to consider are the costs of contact precautions and disinfecting patient rooms. The ADI model assumes full environmental cleaning of rooms occupied by patients in *C*_*S*_ as well.

A full capacity of 21 patients at all times is assumed. Patients have an average stay of 6 weeks, which is extended if a patient obtains an CDI. Simulations were run in MATLAB R2021a 100 times for each scenario for 10 years to calculate yearly averages. A 1-year transient is ignored to obtain steady-state values to compute the averages.

### Parameterization

**Fig. 3.**
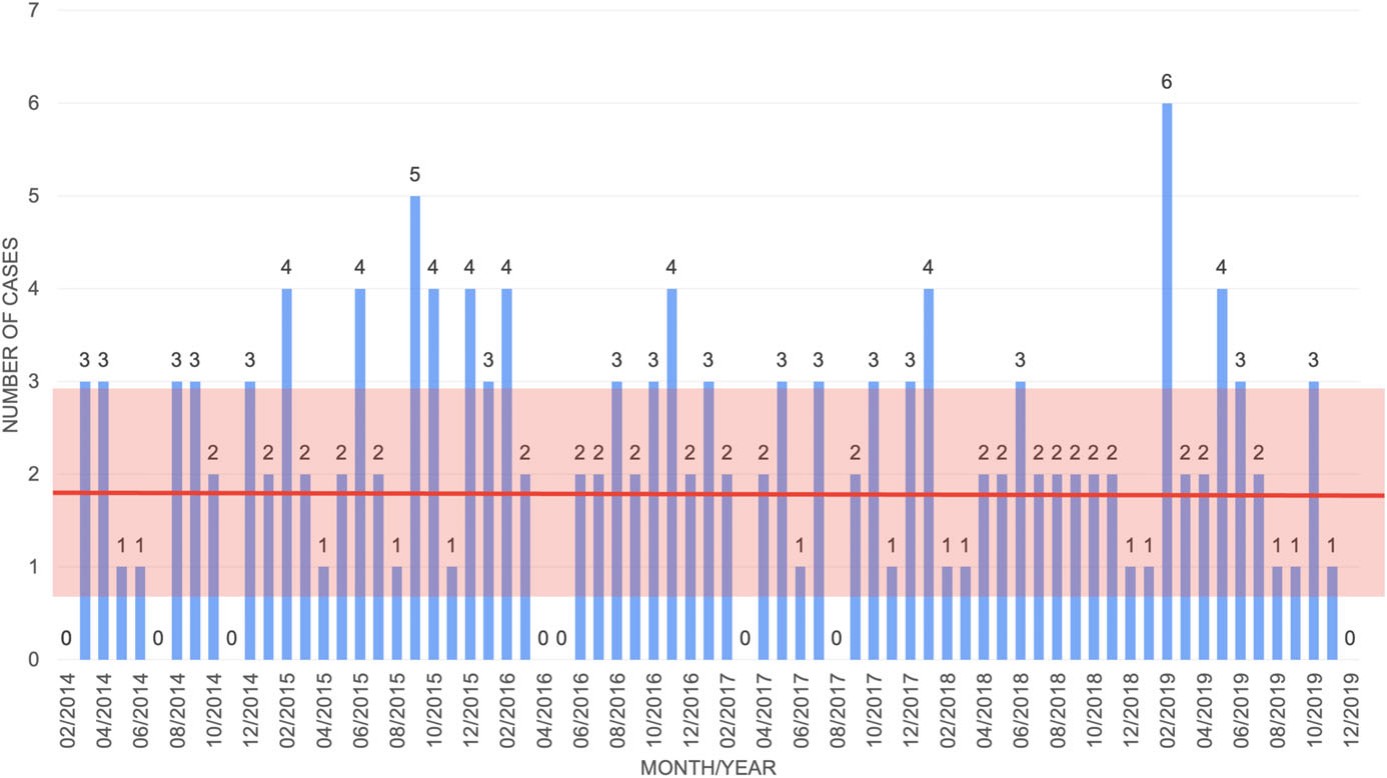
Monthly *C. difficile* data from VCU Medical Center BMT unit from February 2014 to December 2019. The bars represent the monthly cases; the thick red line represents the overall average number of cases; and the red box represents 1 standard deviation above and below the mean.

The parameters used in the simulations are shown in Supplementary Table 1 (online). It has been estimated that ∼14.9% of the general population is colonized with *C. difficile*.^13^ Patients upon admission enter into the *C*_*N*_ or *C*_*S*_ class, with the remaining 85.1%, are assumed to be admitted into *S*. Patients are immunocompromised, and they are given antibiotics for suspected or confirmed infections.^18^ These patients typically receive antibiotics within the first 6 days of their hospital stay; thus, the daily probability of being prescribed antibiotics is *λ* = 1*/*6. If a patient develops CDI, a 10-day course of antibiotics is used to treat the infection, so *γ* = 1*/*10, the daily recovery rate of CDI.^19^ The remaining parameters are estimated based upon CDI data from the BMT unit within VCU Medical Center from February 2014 until December 2019 (77 months) (Fig. 3). The data had a yearly average of 25.29 infections, with standard deviation of 4.19 infections.

**Fig. 4.**
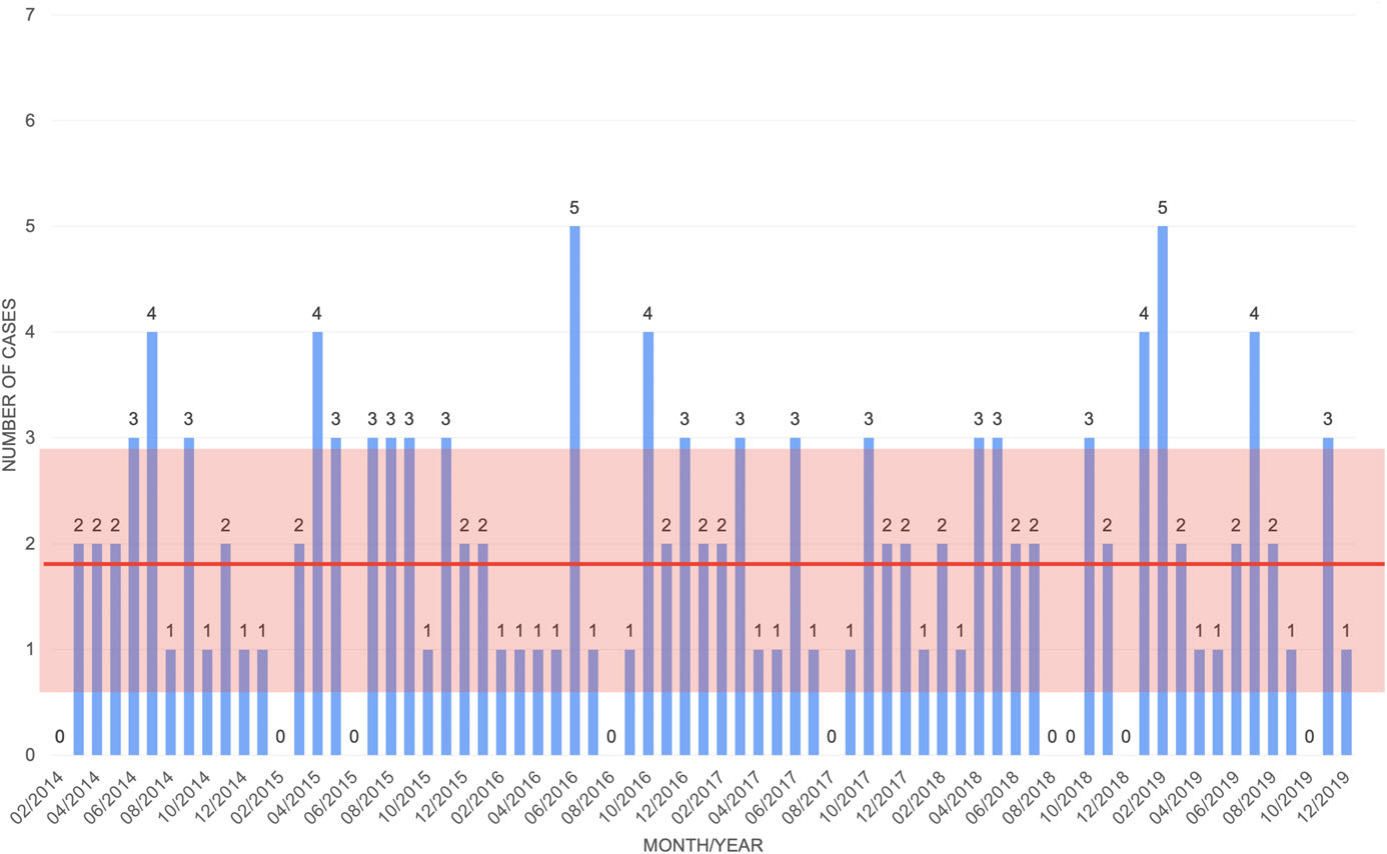
Simulated monthly data with non-ADI model. The blue bars represent the monthly cases; the thick red line represents the overall average number of cases; and the red box represents 1 standard deviation above and below the mean.

The averages taken over 100 simulations with estimated parameters in the non-ADI model agree with the real data, as confirmed by a 2-sample *t* test under 99% confidence. After the simulations were run, a random sample of 77 months was taken using the *datasample* method in MATLAB R2021a. An F test was conducted at 99% confidence to test whether the variances between the simulated data and the real data were the same. The null hypothesis that the variances were the same was not rejected, so a 2-sample *t* test was conducted (Fig. 4).

## Results

**Fig. 5.**
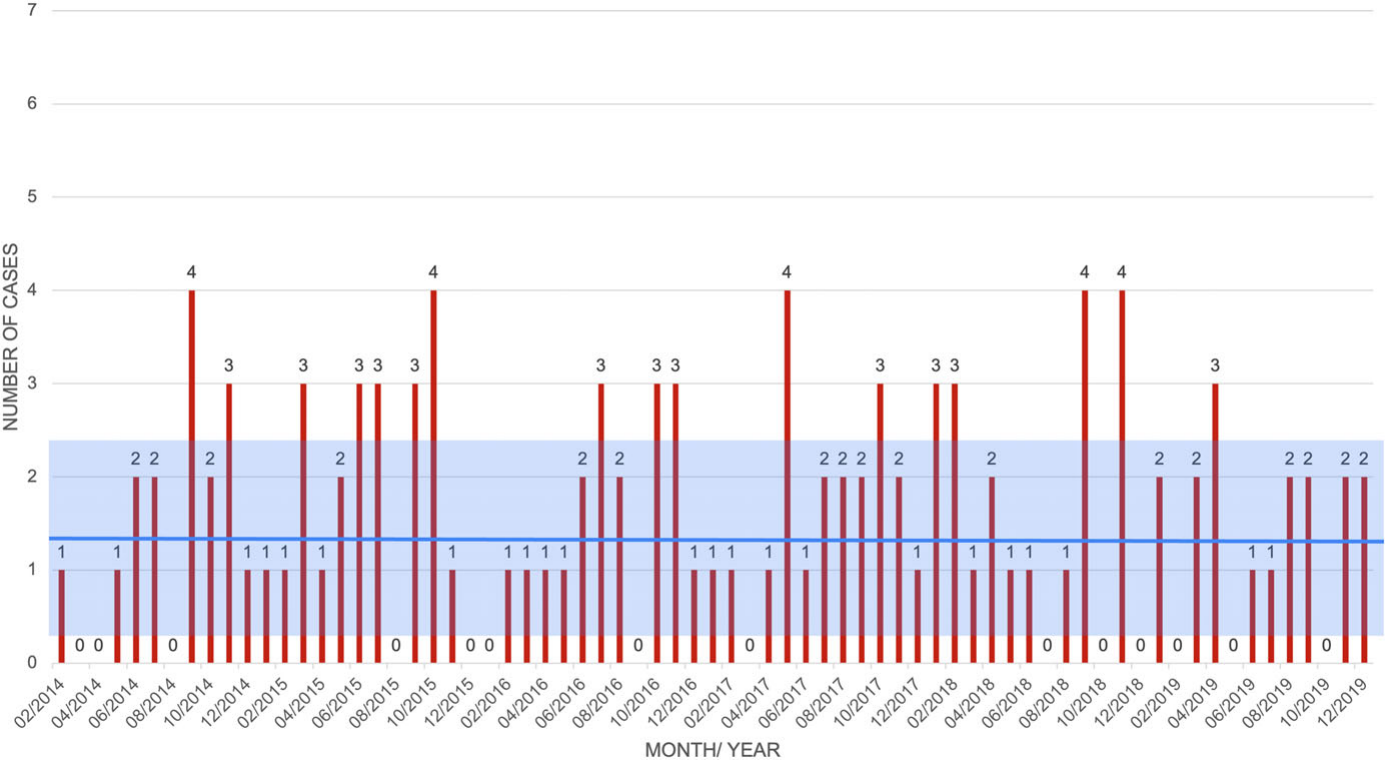
Simulated monthly data with ADI model. The red bars represent the monthly cases; the thick blue line represents the overall average number of cases; and the blue box represents 1 standard deviation above and below the mean.

With ADI implemented, there is an estimated 24.5% decrease in the total number of infections between the non-ADI model and the ADI model (Fig. 5 and Supplementary Table 2 online). The number of community-acquired infections did not change because ADI helps prevent hospital-onset cases of CDI and not CDI cases from those who are already colonized before entering the hospital. This model showed a reduction of 6.2 CDIs per year; a 84.11% reduction in hospital-acquired cases. The reduction in cases of CDI from the ADI model compared to the data are statistically significantly different under 99% confidence in a 2-sample *t* test.

For all cases of CDI, we used the excess hospital cost attributable to a new case of CDI to be $12,313.^20^ These costs include the cost of testing and the costs of treating a case of CDI. The average of 25.29 infections per year results in $311,395.77 spent on treating patients with CDI (Supplementary Table 2 online). The non-ADI model had an average of 25.24 infections per year, which resulted in $310,981.19 spent on treating patients with CDI. When the number of infections was reduced to an average of 19.05 in the ADI model, the total cost for treating patients with CDI decreased to $243,382.80. This decrease represents $67,598.38 saved by preventing CDI with ADI.

## Discussion

We quantified the impact of implementing active detection and isolation in a BMT unit utilizing an agent-base model with 8 patient states. We estimated the number of cases of CDI and the associated costs. The model generated a data set statistically similar to the data provided by VCU Medical Center. The data have a yearly average of 25.29 infections and a yearly standard deviation of 4.19 infections. Upon implementing ADI, we found a 25% reduction, on average, in total cases of CDI per year. Additionally, there is an 84% decrease in hospital-acquired cases alone. We noted the decrease is because of ADI and not due to reclassification, as did Barket et al.^7^

Modeling patients in the BMT unit is challenging due to the varying underlying conditions that the patients are in, the variable, long length of stays and the high rate of antibiotic prescription.^7^ Cases of CDI may occur in outbreaks due to a contaminated environment or may be isolated cases from those that are colonized.

ADI reduces hospital-acquired cases of CDI due to the reduction of spore shedding by infectious patients through the implementation of contact precautions on all known cases of colonization and active infection. Community-acquired colonization identified by ADI can still result in a CDI, even with the additional contact precautions.

These results agree with those of other studies of implementing ADI in the BMT unit showing that ADI could reduce hospital-acquired cases of CDI. We showed a 84% decrease in hospital-acquired cases of CDI when ADI was implemented. An article published in 2017 reported a similar reduction of 83% in a study of the impact of ADI on CDI cases specifically in the BMT unit at a hospital in Madison, Wisconsin. Preintervention and postintervention data showed that 10% of BMT patients were tested for *C. difficile* upon admission before screening was implemented, which increased to 75% upon implementation.^7^

Another study published in 2014 focused on implementing ADI; their results showed a 25% reduction in hospital-acquired cases of CDI with ADI. This study also included 6 medicine wards within a hospital with 2 strains of *C. difficile*. Their data showed that 58% of CDI cases were hospital acquired. After using an agent-based model of ADI, the mean number of hospital-acquired CDI cases were reduced by 25%. However, this study was not conducted in a BMT unit.^17^

This model can answer more questions about preventing cases of CDI from occurring by including testing accuracy. In this study, we did not consider the effect of testing accuracy of CDI; instead, we assumed that testing of CDI and colonization was 100% accurate. If this was not the case, then patient transitions would be more complicated as well as the resulting dynamics.

New costs are associated with implementing active detection and isolation. These costs might be too high to implement hospital wide. However, we make a case for implementing ADI locally in hospital wards with highly vulnerable patients. The BMT ward does have vulnerable patients, and our calculations show that the impact of ADI in reducing hospital-acquired CDI would result in a net savings in healthcare costs.

Unfortunately, community-acquired infections in some communities are inevitable due to a high percentage of people colonized with *C. difficile.* In this research we assumed that ∼14.9% of the population were already colonized with *C. difficile*.^13^ However, as expected, implementing ADI reduces the number of hospital-acquired infections due to contact precautions placed on colonized patients, and the contribution of spores to the environment is reduced. When the environment is less contaminated, patients are less likely to acquire *C. difficile*.

Overall, these models tracked the status of patients in the BMT unit to determine whether patients develop CDI, with and without ADI in place. We broke down the cases of CDI into hospital-acquired and community-acquired cases to quantify the impact of ADI on the reduction of hospital-acquired cases specifically. Given the high cost of a case of CDI and the relatively low cost of a PCR test, this study supports the implementation of ADI from a cost-savings perspective.

## Supplementary material

For supplementary material accompanying this paper visit https://doi.org/10.1017/ice.2023.37. click here to view supplementary material
