# Supplementary material for: Estimating the effect of active detection and isolation on Clostridioides difficile infections in a bone marrow transplant unit
Source: Infect Control Hosp Epidemiol. 2023 Mar 13;44(10):1614–9. doi: 10.1017/ice.2023.37 (PMC10587385; doi:10.1017/ice.2023.37)
Supplement: Supplementary file 1 [file S0899823X23000375sup001.docx]

**Supplementary Materials**

| Parameter symbol | Parameter description | Parameter value | Source |
| --- | --- | --- | --- |
| *αI_N_* | Contribution of spores by patients in the *I_N_* state | 2 | Estimated |
| *b* | Proportion of patients admitted as susceptible | 0.800 | [20] |
| *η* | Environment coefficient | 1000 | Estimated |
| *ψ* | Environment impact scalar | 6 | Estimated |
| *γ* | Rate of recovery | 0.100 | [19] |
| *λ* | Rate of antibiotic prescription | 0.167 | [22] |
| *σ_S_* | Rate of susceptible patients becoming colonized with *C. diff* from the environment | 0.010 | Estimated |
| *σS_A_* | Rate of susceptible patients on antibiotics becoming colonized  with *C. diff* | 0.050 | Estimated |
| *θC_A_* | Rate of colonized patients on antibiotics becoming symptomatic | 0.017 | Estimated |
| *θC_H_* | Rate of colonized patients from the environment becoming symptomatic | 0.100 | Estimated |
| *θC_N_* | Rate of admitted colonized patients becoming symptomatic in non-ADI model | 0.017 | Estimated |
| *θC_S_* | Rate of admitted colonized patients becoming symptomatic in ADI-model | 0.017 | Estimated |

**Table 1.** Parameter values used in the simulations. Estimated parameters come from data from the BMT Unit at VCU Medical Center from February 2014 - December 2019.

|  | Data | non-ADI model | ADI model |
| --- | --- | --- | --- |
| Percent of infections  that are community-acquired | 69% | 70% | 94% |
| Percent of infections  that are hospital acquired | 31% | 30% | 6% |
| Average number of  community-acquired  infections per year | 17.25 | 17.89 | 17.99 |
| Average number of  hospital-acquired  infections per year | 7.75 | 7.62 | 1.19 |
| Average number of  infections per year | 25.29 | 25.60 | 19.18 |
| Number of tests per year | – | 25.60 | 195.46 |
| Number of additional  patients placed under  contact precautions per year | – | – | 17 |
| Number of additional room  to disinfect upon discharge | – | – | 17 |
| Cost of infections per year | $348,478.75 | $352,750.34 | $264,287.17 |
| Cost of tests per year | – | $200.19 | $1,329.40 |
| Cost of additional contact  precautions per year | – | – | $1,845.35 |
| Cost of additional terminal  cleaning per year | – | – | $9,616.73 |
| Total cost per year | – | $352,950.53 | $277,277.75 |

**Table 2.** Simulation results comparing the ADI model with the non-ADI model. We also include the data averages from the BMT unit at VCU Medical Center from February 2014 - December 2019.
